# Cytokine Contents in Chronic Lymphocytic Leukemia: Association with ZAP70 Expression

**DOI:** 10.4274/tjh.2014.0469

**Published:** 2016-08-19

**Authors:** Nilgün Işıksaçan, Suzan Çınar, Esin Aktaş Çetin, Melih Aktan, Günnur Deniz

**Affiliations:** 1 Bakırköy Dr. Sadi Konuk Training and Research Hospital, Central Laboratory, İstanbul, Turkey; 2 İstanbul University Institute of Experimental Medicine, Department of Immunology, İstanbul, Turkey; 3 İstanbul University İstanbul Faculty of Medicine, Department of Internal Medicine, Division of Hematology, İstanbul, Turkey

**Keywords:** ZAP70, Interleukin-4, Interferon gamma, T cells, B cells, Chronic lymphocytic leukemia

## Abstract

**Objective:**

Chronic lymphocytic leukemia (CLL) is a disease that shows varying clinical progression, and expression of the protein tyrosine kinase ZAP70 has been described as a very valuable prognostic factor. Patients with ZAP70 positivity are characterized by worse clinical course and significantly shorter progression-free and overall survival. In this study, intracytoplasmic interferon gamma (IFN-γ) and interleukin-4 (IL-4) content of T, B, and CLL cells in CLL patients and their correlations with Rai staging and ZAP70 positivity were investigated.

**Materials and Methods:**

CLL patients newly diagnosed or in follow-up at the İstanbul University İstanbul Medical Faculty Hematology Department were included in this study. These patients were classified according to Rai staging and ZAP70 expression. IL-4, IFN-γ, and ZAP70 expressions in peripheral blood T, B, and CLL cells were measured by four-color flow cytometry.

**Results:**

There was a statistically significant correlation between advanced disease and ZAP70 positivity. IL-4-secreting T cells were significantly increased; however, IFN-γ secretion was significantly decreased in CLL patients compared to healthy individuals, whereas IL-4-secreting B cells were significantly diminished in contrast to T cells.

**Conclusion:**

These findings suggest damage in the cellular immunity and that IL-4 might lead to many complications and may be important in disease progression.

## Abstract (tr)

**Amaç:**

Kronik lenfositik lösemi (KLL) klinik olarak çok farklı seyir gösterebilen bir hastalıktır ve tirozin kinaz ZAP70’in ekspresyonu, çok değerli bir prognostik faktör olarak tanımlanmıştır. ZAP70 pozitif hastalar, kötü klinik seyir, belirgin olarak kısa hastalıksız geçen süre ve düşük sağkalım oranı ile karakterizedir. Bu çalışmada KLL hastalarının T, B ve KLL hücrelerinde intrasitoplazmik interferon gama (IFN-y) ve interlökin-4 (IL-4) içeriği, Rai evrelemesi ve ZAP70 pozitifliği arasındaki korelasyon araştırılmıştır.

**Gereç ve Yöntemler:**

Bu çalışmaya İstanbul Üniversitesi İstanbul Tıp Fakültesi Hematoloji Kliniği’nde tanısı yeni konan veya takip edilmekte olan KLL hastaları dahil edilmiştir. Bu hastalar Rai evreleme sistemi ve ZAP70 ekspresyonlarına göre sınıflandırılmıştır. Periferik kan T, B ve KLL hücrelerinin IL-4, IFN-g ve ZAP70 ekspresyonları 4 renkli flow sitometri ile ölçülmüştür.

**Bulgular:**

Hastalığın evresindeki ilerleme ile ZAP70 pozitifliği arasında istatistiksel olarak anlamlı bir korelasyon bulunmaktadır. KLL hastalarının sağlıklı bireylerle kıyaslandığında IL-4 salgılayan T hücreleri sayısı anlamlı olarak artmış, ancak IFN-g salgıları anlamlı olarak azalmış, IL-4 salgılayan B hücre sayısı ise T hücrelerinin aksine anlamlı olarak azalmıştır.

**Sonuç:**

Bu bulgular, hücresel immünitede hasarın olabileceğini ve IL-4’ün birçok komplikasyonlara yol açarak hastalığın ilerlemesinde önemli olabileceğini düşündürtmektedir.

## INTRODUCTION

Chronic lymphocytic leukemia (CLL) is a disease that shows varying clinical progression. The Rai and Binet classification systems are useful to predict treatment requirements and survival rates for CLL patients, but current classifications fail to distinguish patients who may develop aggressive disease [1,2,3,4]. The somatic mutations in the immunoglobulin heavy chain variable (IGVH) region are very valuable prognostic factors and, in CLL cases, IGVH-mutated patients have a better clinical outcome while nonmutated patients have a poorer prognosis and impaired response to chemotherapy [5,6,7].

The protein tyrosine kinase zeta-associated protein (ZAP70) is a protein tyrosine kinase in the T cell receptor signal transduction system that can be detected in CLL cells. Studies have pointed out a correlation between ZAP70 expression in CLL cells and IGVH status, and showed that ZAP70 positive patients have an aggressive course, an immediate treatment requirement, longer therapy time, and lower survival rates [8,9,10]. Expression of ZAP70 has been described as a very valuable prognostic factor [2,8].

Many researchers have recently examined the cytokine content of the T cells in CLL patients and emphasized the significance of the T cell activity in the prognosis of the disease [11,12]. Interleukin-4 (IL-4) production by T cells has been shown to significantly increase in patients with progressive disease [11,12]. In light of these data, it was suggested that in CLL, the type 1 T cell cytokine profile is converted to the type 2 T cell profile in the advanced stages of the disease [13]. The aim of this study was to evaluate the expression of ZAP70 changing during disease progression, the intracellular interferon gamma (IFN-γ) and IL-4 content of T and B lymphocytes and the CLL cell subset (CD5+CD19+) in CLL patients and healthy subjects, and ZAP70 correlation with cytokine production.

## MATERIALS AND METHODS

### Study Population

Twenty-eight patients in follow-up at the İstanbul University İstanbul Medical Faculty Hematology Department were included in the study. Patients were diagnosed according to the CLL diagnosis criteria published in 1996 by a study group supported by the National Cancer Institute. Clinical data and follow-up files of all the patients included in the study were gathered. All patients received written information about the study, including ethics committee approval, before the study was initiated.

Twenty-eight CLL patients (18 males, 10 females) and 10 healthy individuals (3 males, 7 females) were involved in this study (Table 1). The ZAP70 expression was measured for all CLL cases and cytokine levels were measured for 12 CLL cases and the 10 healthy volunteers. The ages of the patients were between 36 and 81 (59±11) years, and the mean values were 60±12 years for males and 56±7 years for females.

### Peripheral Blood Mononuclear Cell Isolation and Flow Cytometric Analyses of Lymphocyte Subsets

Samples were processed using a whole-blood lysis method to analyze lymphocyte subsets. Heparinized blood samples were collected from patient and healthy donors and stained with anti-CD19-fluorescein isothiocyanate (FITC), anti-CD3-allophycocyanin (APC), anti-CD5-TRI-COLOR (TC), anti-CD45-FITC, anti-CD14-phycoerythrin (PE), anti-CD23-PE, anti-CD38-PE, and PE, FITC, TC, or APC conjugated isotype control (IC) monoclonal antibodies (mAbs) (all from Caltag Laboratories, Austria) for 30 min at room temperature. Lysis was performed with FACS Lysing Solution (BD Biosciences, USA). After washing cells with phosphate-buffered saline (PBS), stained cells were fixed in 1% paraformaldehyde and the cells were analyzed with BD FACSCalibur with CellQuest Software (BD Biosciences).

For the intracytoplasmic ZAP70 expression, the cells were labeled with anti-CD19-FITC and anti-CD5-TC mAbs (Caltag Laboratories) for 30 min at room temperature. After the lysing period, cells were washed with PBS and were fixed and permeabilized with paraformaldehyde/saponin solution (Cytofix&Cytoperm Kit, BD Biosciences), and then were stained with PE-conjugated-IgG1 or PE-labeled ZAP70 (Caltag Laboratories) mAbs for 30 min at room temperature and analyzed by flow cytometry. Each analysis was performed using at least 5000 cells gated in the region of the B cell population, and the cells were analyzed with BD FACSCalibur with CellQuest Software (BD Biosciences). ZAP70 expression was investigated in CLL patients for positivity; 20% was used as the cut-off [9].

**Figure 1:**
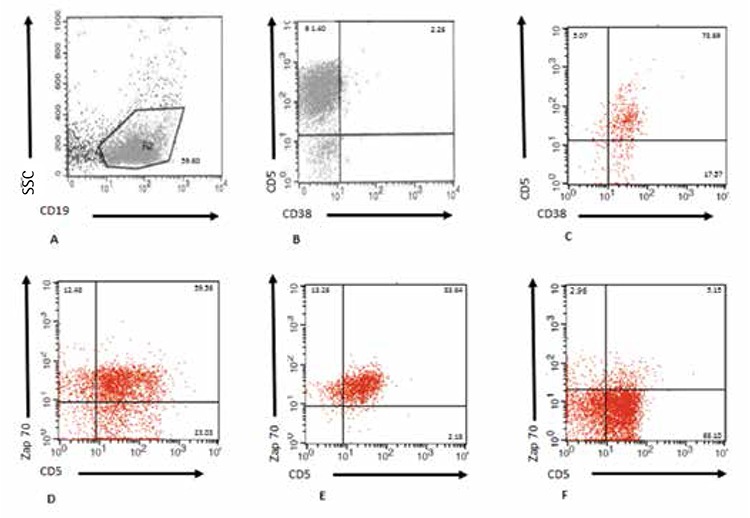
Gating strategy of chronic lymphocytic leukemia. Peripheral blood mononuclear cells from chronic lymphocytic leukemia patients (n=28) were stained for CD5, CD19, CD38, and ZAP70 mAbs and analyzed by flow cytometry. CD19+ cells were gated versus SSC (A). Representative dot-plot analyses for the expression of a patient negative (B) and positive (C) for CD38 with CD5+ in CD19+ cells are shown. In the CD19+ B cell population, CD5+ZAP70+ (D, E) and CD5+ZAP70- (F) plots from three different patients with chronic lymphocytic leukemia are also indicated. The numbers indicate the proportion of cells positive for indicated markers.

CD19+ cells were analyzed for the expression of CD5, CD38, and ZAP70 (Figure 1A). Most of the CD19+ B cells expressed CD5; however, there was only one patient expressing both CD5 and CD38 (Figures 1B and 1C, respectively). Expression of CD5 showed heterogeneity among the patients. Intracytoplasmic ZAP70 together with CD5 was expressed in different levels in CD19+ B cells (Figures 1D, 1E, and 1F).

### Intracytoplasmic Cytokine Staining

Peripheral blood mononuclear cells (PBMCs) were separated using Ficoll-Hypaque (Sigma Chem. Co., USA) density gradient centrifugation, and cells were washed twice in Hank’s balanced salt solution. The cells were finally adjusted to a final concentration of 1x106 cells/mL in complete RPMI-1640 medium (Sigma Chem. Co.) supplemented with 10% heat-inactivated fetal calf serum, penicillin (100 U/mL), streptomycin (100 mg/mL), gentamicin (50 mg/mL), and 50 µM 2-mercaptoethanol. Freshly purified PBMCs were washed and 1x106 cells/mL were stimulated for 18 h by a combination of phorbol ester, phorbol-12-myristate-13-acetate (50 ng/mL), and Ca2+ ionophore (ionomycin, 250 ng/mL) in a 24-well round-bottom plate (all from Sigma Chem. Co.). The combination of these two stimuli was used to achieve the strongest stimulus for intracytoplasmic cytokine secretion. Monensin (Sigma Chem. Co.) was added at a final concentration of 1 µM to the cultures in the final 4 h.

After incubation, PBMCs were washed with PBS solution and stained with anti-CD19-PE and anti-CD3-APC mAbs for 30 min (Caltag Laboratories) for determining the cytokine secretion of T and B cells. Cells were washed with PBS and then fixed and permeabilized with paraformaldehyde/saponin solution (Cytofix&Cytoperm Kit, BD Biosciences). After washing, the cells were stained with FITC conjugated IC (IgG1), anti-IL-4-FITC, and anti-IFN-γ-FITC (Caltag Laboratories) mAbs for 30 min at room temperature. After washing, cells were resuspended in 1% paraformaldehyde (Sigma Chem. Co.) and analyzed by flow cytometry. Each sample was acquired with a BD FACSCalibur (BD Biosciences) and analyzed with the instrument’s operating software, CellQuest (BD Biosciences).

### Statistical Analysis

Statistical analysis was performed using a standard nonparametric Mann-Whitney U test using SPSS 17.0 for Windows. The results are presented as median values and p<0.05 was accepted as the statistical significance level.

## RESULTS

### Increased Expression of ZAP70 in Chronic Lymphocytic Leukemia Patients

**Figure 2:**
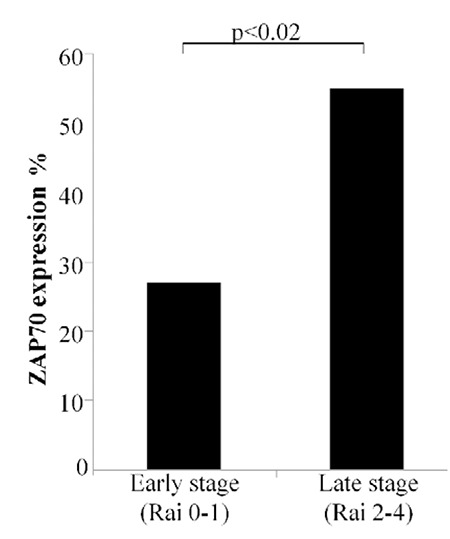
Expression of ZAP70+ cells according to Rai classification system. Chronic lymphocytic leukemia patients were divided into two groups according to the Rai staging system. Summarized data showing the percentages of ZAP70 expression in chronic lymphocytic leukemia patients and significant differences are indicated. ZAP70 expression was 23.5% (3.02-85.55) [median (min-max)] in Rai 0-1 as the early stage and 51.84% (6.81-99.05) in Rai 2-4 as the late stage among 28 chronic lymphocytic leukemia patients.

ZAP70 levels in CD19+CD5+ CLL patients did not show any differences according to sex (p>0.05). Seventeen patients were positive for ZAP70; 8 of them were Rai 0-1 and 9 of them were Rai 2-4. However, 7 patients negative for ZAP70 were Rai 0-1 and 4 patients for Rai 2-4. When patients are divided into two groups according to Rai staging system, the first group includes 15 patients in stages 0 and 1, and the second group includes 13 patients in stages 2 and 4. The difference between ZAP70 expression in the first and second Rai groups was statistically significant (p<0.02) (Figure 2).

### CD38 Expression with ZAP70

Increase in CD38 expression correlates with increased lymphocyte proliferation and disease progression. CD38 expression is important in prognosis when combined with ZAP70. All patients were analyzed for CD38 expression, and an expression level of ≥30% was accepted as positive [14]. In CD19+ B cell populations, 26 patients were CD5+CD38-; however, only one patient was CD5+CD38+ and also ZAP70 positive (data not shown).

### Increased IL-4 and Decreased IFN-γ Levels in Chronic Lymphocytic Leukemia Patients

In the first step, CLL patients were divided into two main groups according to their CD19, CD5, and ZAP70 expression (CD19+CD5+ZAP+ and CD19+CD5+ZAP-). Secondly, CD3+ T, CD19+ B, and CD19+CD5+ cells of CD19+CD5+ZAP+ and CD19+CD5+ZAP- CLL patients were evaluated for their intracellular IL-4 and IFN-γ contents.

**Figure 3:**
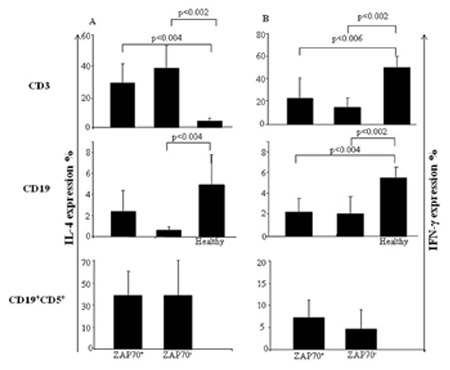
Comparison of intracellular IFN-γ and IL-4 production of CD3+ T, CD19+ B, and CD19+CD5+ cells between ZAP70+ and ZAP70+ chronic lymphocytic leukemia patients and healthy subjects. Intracellular cytokine levels were measured in stimulated peripheral blood mononuclear cells obtained from chronic lymphocytic leukemia patients (n=28) and healthy individuals (n=10). IL-4 and IFN-γ secretions were analyzed by flow cytometry. Results are shown as median (range) deviation.

IL-4 levels of CD3+ T cells in ZAP70+ and ZAP70- patients were significantly increased in both groups compared to healthy individuals (p<0.004 and p<0.002, respectively). In contrast, IFN-γ secretion of CD3+ T cells was significantly decreased in both groups compared to healthy individuals (p<0.006 and p<0.002, respectively). However, IL-4 and IFN-γ levels did not differ in CD3+ T cells of ZAP+ and ZAP- CLL patients (Figures 3A and 3B).

IL-4 levels of CD19+ B cells showed significant decreases in ZAP70- CLL patients compared to healthy subjects (p<0.004), whereas IL-4 levels between ZAP70+ and ZAP70- patients did not show any difference. IFN-γ levels of CD19+ B cells in ZAP70+ patients did not show any difference in comparison to ZAP70- patients, but both patient groups had significantly lower IFN-γ levels than healthy subjects (p<0.04 and p<0.02, respectively) (Figures 3A and 3B).

There was no significant difference in IL-4 and IFN-γ expression in CD19+CD5+ cells in ZAP70+ and ZAP70- patients (Figures 3A and 3B). The IL-4/IFN-γ ratios in CD3+ T, CD19+, and CD19+CD5+ B cells were analyzed and no significant difference for the expression of ZAP70 was observed (data not shown).

## DISCUSSION

Chronic lymphocytic leukemia is leukemia of small, mature B cells; it mostly affects adults over 65 years of age and it is the most common form of lymphoid malignancy. The Rai and Binet classification systems are useful to predict treatment requirements and survival for CLL patients, but the prognostic value of these classifications is limited in early stage cases. From 30% to 40% of patients in the early stage may develop aggressive disease and die in a short time period [15,16].

It has been shown that for ZAP70 positivity, when a cut-off value of 20% was used, CLL patients could be classified into two groups: those with levels of <20% had increased survival time and decreased chance of disease progression [9]. Two main techniques for measuring ZAP70 by flow cytometry involve comparing ZAP70 expression in B cells either against an isotype control or against ZAP70 expression in T cells [1,17]. In this study, B cell surface marker CD19 and CLL-specific surface marker CD5 were used and ZAP70 expressions in the double-positive CD19+CD5+ cells were evaluated; ZAP70 expressions of T cells were easily excluded. A great deal of information can be extracted about the presence and status of CLL from the ZAP70 expression in the Rai staging system. The statistically significant difference of ZAP70 expression in stages 2 and 4 compared to early stages with significantly more ZAP70 patients in Rai stages 2 and 4 in comparison to Rai stages 0 and 1 is consistent with the literature and was evaluated as a marker of poor diagnosis.

CD38 is a glycoprotein found on hematopoietic cells including B cells; it is a marker of cell activation and also functions in proliferation and activation [18]. Association between ZAP70 as an IGVH mutation status and CD38 has been investigated [19]. In contrast to high expression of ZAP70 (53%), decreased CD38 expression in CLL patients might be related to the variability during the disease. The combination of ZAP70 and CD38 expression gives complementary prognostic information, whereas this analysis allows us to identify three isolated B-CLL patient subgroups with good, intermediate, and poor prognosis to decide how to treat them, especially in early clinical stages of the disease. ZAP70 expression is stable during the course of the disease and it can be relatively easily measured in diagnostic laboratories with flow cytometers in contrast to altered CD38 expression, which is known as an independent prognostic factor in CLL [20].

It has been found that age and stage at presentation predict prognosis in survival in newly diagnosed patients with CLL [21]. Studies about age at diagnosis and ZAP70 levels in CLL patients showed no association. Similar to recent findings, there was no relation between ZAP70 and sex in our study [22,23,24].

CLL cells are nondividing monoclonal neoplastic CD5+ B cells and they are arrested at the G0 phase of the cell cycle. Failure to induce apoptosis plays a role in accumulation of the leukemic cells rather than cell proliferation. CD19+CD5+ CLL cells are greatly influenced by T cell-derived cytokines such as IL-1-alpha, IL-1-beta, IL-6, IL-7, IL-8, IL-10, IL-13, IFN-γ, and transforming growth factor (TGF)-β, which mediate interactions between B and T cells [25,26]. Apoptosis, the programmed cell death, can be activated in different cell types in response to a number of physiologically relevant stimuli. CLL CD5+ B cells are arrested in G0 and display enhanced survival in vivo, whereas they undergo spontaneous apoptosis in vitro [27]. Changes of the cytokine network in malignant cells and T cells due to various reasons may lead to expansion of the B cell clones. The formation of a new cytokine network may govern cell proliferation and apoptosis in CLL patients.

Studies indicate the development of secondary neoplasias, infectious complications, and autoimmune disorders in advanced disease due to T cell defects and alterations in the cytokine content [12]. IL-4 and IFN-γ are suggested to regulate leukocyte adhesion molecule-1, whereas they take part in the interaction of tumor cells and immune effector cells, and T cell dysfunction in the advanced stage of the disease; therefore, altered cytokine profiles may affect the pathogenesis of the disease [28]. The cytokine microenvironment plays a critical role for Th1 and Th2 polarization, and B-CLL cells express several cytokines such as TGF, IL-10, and IL-4. It is suggested that in patients with B-CLL, with or without ZAP-70 overexpression, distinct cytokine patterns are triggered. It is possible that the IL-4, IL-10, and tumor necrosis factor-α cytokine profile of ZAP-70+ CD4 lymphocytes might favor B-CLL cell growth and survival and, in contrast, the IFN-γ and IL-2 profile in CD4 lymphocytes from ZAP-70- B-CLL patients might promote an antitumor cytotoxic CD8+ T lymphocyte and NK cell response (29).

Our observations of high levels of IL-4 but diminished IFN-γ content of CD3+ T cells in CLL patients suggest that the Th1 cytokine profile turns into the Th2 cytokine profile in CLL patients. Several studies indicate the dominancy of Th1 in the early stage of the disease, but in the advanced stage Th1 decreases and Th2 increases. Th1/Th2 ratio is lower in the advanced stage of the disease than in early stages [12] and it has been suggested that the transformation of Th1 to Th2 is the reason for disease progression [13].

Our findings support the other recent findings, and the transformation of Th1 to Th2 might be related to ZAP70 expression. The cause of Th1 and Th2 count alteration in cancer immunity is not clear; however, the shifting of the Th1 cytokine profile to Th2 seems to be associated with ZAP70. The role of cytokines in CLL is a new topic and it should be analyzed further. High levels of ZAP70 expression and IL-4 secretion might be indicative of poor prognosis, and increased levels of IL-4 may be caused by infections and autoimmune diseases that accompany the disease. On the other hand, IL-4 can lead to the release of the growth factors that are increased in the cytoplasm of CLL cells.

## Ethics

Ethics Committee Approval: The study protocol was approved by the local ethical committee; Informed Consent: It was taken.

## Figures and Tables

**Table 1:**
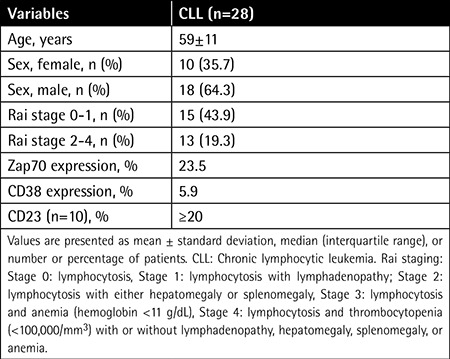
Demographic and clinical characteristics of patient group.
